# Proteomics Reveals an Increase in the Abundance of Glycolytic and Ethanolic Fermentation Enzymes in Developing Sugarcane Culms During Sucrose Accumulation

**DOI:** 10.3389/fpls.2021.716964

**Published:** 2021-09-30

**Authors:** Luis Felipe Boaretto, Mônica Teresa Veneziano Labate, Livia Maria Franceschini, Thais Regiani Cataldi, Ilara Gabriela F. Budzinski, Fabricio Edgar de Moraes, Carlos Alberto Labate

**Affiliations:** Laboratório Max Feffer de Genética de Plantas, Departamento de Genética, Escola Superior de Agricultura “Luiz de Queiroz”, Universidade de São Paulo, São Paulo, Brazil

**Keywords:** *Saccharum* spp., hypoxia, sucrose accumulation, glycolysis, ethanolic fermentation

## Abstract

Sugarcane is an economically important crop contributing to the sugar and ethanol production of the world with 80 and 40%, respectively. Despite its importance as the main crop for sugar production, the mechanisms involved in the regulation of sucrose accumulation in sugarcane culms are still poorly understood. The aim of this work was to compare the quantitative changes of proteins in juvenile and maturing internodes at three stages of plant development. Label-free shotgun proteomics was used for protein profiling and quantification in internodes 5 (I_5_) and 9 (I_9_) of 4-, 7-, and 10-month-old-plants (4M, 7M, and 10M, respectively). The I_9_/I_5_ ratio was used to assess the differences in the abundance of common proteins at each stage of internode development. I_9_ of 4M plants showed statistically significant increases in the abundance of several enzymes of the glycolytic pathway and proteoforms of alcohol dehydrogenase (ADH) and pyruvate decarboxylase (PDC). The changes in content of the enzymes were followed by major increases of proteins related to O_2_ transport like hemoglobin 2, ROS scavenging enzymes, and enzymes involved in the ascorbate/glutatione system. Besides, intermediates from tricarboxylic acid cycle (TCA) were reduced in I_9_-4M, indicating that the increase in abundance of several enzymes involved in glycolysis, pentose phosphate cycle, and TCA, might be responsible for higher metabolic flux, reducing its metabolites content. The results observed in I_9_-4M indicate that hypoxia might be the main cause of the increased flux of glycolysis and ethanolic fermentation to supply ATP and reducing power for plant growth, mitigating the reduction in mitochondrial respiration due to the low oxygen availability inside the culm. As the plant matured and sucrose accumulated to high levels in the culms, the proteins involved in glycolysis, ethanolic fermentation, and primary carbon metabolism were significantly reduced.

## Introduction

Currently, Brazil has 8.8 million hectares of cultivated sugarcane, producing ~642.7 million tons of sugarcane in the 2019/20 crop season (CONAB, 2020). The growing demand for sugar and ethanol production in recent years has prompted a wide interest of plant breeders and biotechnologists to enhance the capacity of sucrose accumulation in sugarcane. In the last 50 years, sugarcane breeders achieved significant progress in increasing productivity through conventional breeding, mainly by intercrossing elite cultivars (Lakshmanan et al., 2005). However, to enhance the gains in sugar content, the necessity of an integrated approach, combining conventional, and molecular breeding strategies is becoming clear. The main hurdle for the success of such an approach is the complex genetic basis of sugarcane, which has a polyploidy genome, with many varieties being aneuploid. Today, the majority of the planted sugarcane cultivars are complex hybrids derived mainly from crosses between *Saccharum officinarum* and *Saccharum spontaneum* (Lakshmanan et al., 2005).

The mechanisms involved in the regulation of sucrose accumulation in sugarcane culms are still poorly understood. Sucrose can reach high concentrations in the storage tissues of some varieties, up to 650 mM (Hawker, 1985; Welbaum and Meinzer, 1990). For a long time, the process of sucrose accumulation was considered a simple event, related to the photosynthetic production of sucrose in the leaf and translocation through the phloem tissue to the culms (Moore, 1995). However, since the 1970s, several studies have focused on discovering the mechanisms related to the process of sucrose metabolism in the parenchyma cells of culms (Glasziou and Gayler, 1972). Partitioning of sugars among different tissues depends on specific control points along the transport pathways. Thus, knowledge of these points is crucial for a better understanding of the sucrose storage process (Rae et al., 2005).

Sucrose, present in the cytosol of parenchyma cells, is cleaved by neutral invertases, which also play a role in sucrose accumulation and utilization. Different from the products generated by invertases, sucrose synthase (SuSy) degrades sucrose producing UDP-glucose and fructose, which are intermediates used for cell wall biosynthesis and produce energy for growth (Schäfer et al., 2004). SuSy has also been associated with the elongation process of the internodes and is probably involved in sucrose synthesis in young internodes (Verma et al., 2011).

During internode development, the concentration of sucrose was shown to be dependent on the respiratory rate, which decreases by ~50% in mature culms (Whittaker and Botha, 1997). Such changes in respiratory flux along internode maturation may not be linked to sucrose loading, but rather to other physiological limitations such as the exposure of culms to hypoxia (low O_2_ concentration), leading to a major shift in carbon metabolism, as sucrose reaches its maximum accumulation. Therefore, some questions are raised as to how glycolysis, ethanolic fermentation, pentose phosphate, and tricarboxylic acid cycle (TCA) pathways are regulated during internode maturation, as this process is still poorly understood.

Under field conditions, the concomitant occurrence of water stress or low temperature further enhances sucrose accumulation in the culms. The identification of drought-responsive proteins and the changes in their abundance that occurs during sucrose accumulation are, therefore, important to understand the regulation of sucrose accumulation during culm maturation. Sucrose accumulation in the culms is complex and dynamic, and only partially elucidated by examining transcript abundance (Papini-Terzi et al., 2009; Iskandar et al., 2011; Kido et al., 2012).

Limited proteomic information is available about sucrose accumulation in sugarcane culms, and most of the work has focused on a small number of proteins (Jangpromma et al., 2010). In the present study, we used the label-free shotgun proteomics approach to gain a comprehensive and complementary insight into the changes occurring in the proteome of sugarcane internodes of a commercial cultivar during plant development and sucrose accumulation. Primary metabolites from 4M internodes were also analyzed aiming to complement the proteomic data. We observed major changes in the proteins involved in the carbon metabolism during the initial stages of internodes development, with glycolysis and ethanolic fermentation playing an important role in providing energy and reducing power for plant growth. These results play an important role to understand the physiological changes that occur in sugarcane from a juvenile stage to maturation.

## Materials and Methods

### Plant Material and Experimental Conditions

Sugarcane cultivar SP80-3280 was grown in greenhouse conditions at Piracicaba, São Paulo, Brazil (22°43′31″S; 47°38′57″W). Culms with one bud were planted in plastic trays, and after acclimatization for 2 months, nine plants were transferred to 100-L plastic pots, containing soil (latosol). The temperature in the greenhouse was automatically adjusted to 28 ± 1°C and 12/12 h light/dark photoperiod. Plants were watered to maintain soil capacity once a day in the morning. Fertilizer was supplied once a week using a commercial fertilizer (Plant Prod^®^, N 15: P_2_O_5_ 15: K_2_O 30, Plant Products CO. Canada) at the rate of 2 L per pot (4 g/L). The replicates, distributed in a randomized design, were harvested at three stages of development: 4M, 7M, and 10M. Ten-month-old plants were harvested after 10 days of drought stress to increase the sugar content in the culms. During harvest, leaves were excised from the main culm and the internodes 4, 5, 6, 7, 8, and 9 from each plant, separated and peeled from the top toward the base. Each individual internode was frozen in liquid nitrogen and kept at −80°C. Soluble sugars content was determined for all internodes which were also used for proteomic analyses. Three biological replicates from each internode were harvested at each developmental stage.

### Storage Sugar Determination From Apoplast and Symplast by Ion Chromatography

The apoplast fluid from internodes 4, 5, 6, 7, 8, and 9 of 4M, 7M, and 10M plants were extracted by centrifugation of the inner part of each internode after the removal of bark (1.0 cm × 0.5 cm × 0.5 cm) at 480 *g* for 10 min, according to Welbaum and Meinzer (1990), and kept at −20°C till analyses. Symplast soluble sugars were extracted by incubating the internode samples with 10 mL of deionized water at 80°C for 60 min in a water bath. The liquid solution was separated by vacuum filtration. The volume of the solution of soluble sugars was then raised to 200 mL with milliQ water for all samples. The main soluble carbohydrates from apoplast and symplast fluids (glucose, fructose, and sucrose) were quantified by HPLC-PAD ICS 2500 (Dionex, California) equipped with a pulsed amperometric ion detector and a CarboPac™ PA1 anion exchange column. Five millimolars of NaOH solution was used as the mobile phase with a flow rate 0.25 mL/min. Calibration curves were prepared with standard sugars d-(+)-glucose, d-(–)-fructose and sucrose, from Sigma.

### Metabolite Extraction and Derivatization for GC-TOF-MS

Metabolites from I_5_-4M and I_9_-4M were extracted from six biological samples of 4-month-old plants. Following the removal of leaves, the internodes were identified and cut, the bark removed (see Supplementary Figure 2), and the remaining tissue was immediately frozen in liquid nitrogen. Metabolite extraction was carried out as described in Budzinski et al. (2019).

### Metabolite Identification and Statistical Analysis

The ChromaTOF software v. 4.51 (Leco Corp., St. Joseph, USA) was used to perform baseline correction, deconvolution, peak detection, retention time alignment, and library matching, considering similarity ≥700. NIST mass spectral library was used for metabolite identification. Multivariate [partial least squares-discriminant analysis (PLS-DA)] and univariate (*t*-test, FDR-adjusted *p* ≤ 0.05) analysis were done using the MetaboAnalyst 5.0 Software (Xia et al., 2015). To reduce systematic variance and to improve the performance for downstream statistical analysis, data were log-transformed and auto-scaled prior to data analysis. Metabolites detected in at least three replicates in each group (I_5_ or I_9_) and not identified in any replicate of the other group were named “unique” metabolites.

### Protein Extraction and Digestion

Proteins were extracted according to the protocol described by Hurkman and Tanaka (1986), with a few modifications. Samples (1 g) from the I_5_ and I_9_, collected from three individual plants at different ages, were pulverized to a fine powder in liquid nitrogen using mortar and pestle. Samples were homogenized at 4°C for 30 min in 15 mL of extraction buffer (0.5 M TrisHCl, pH 7.5; 0.1 M KCl; 0.05 M EDTA; 0.7 M sucrose; 2% v/v 2-mercaptoethanol; 2 mM PMSF; 1% w/v polyvinylpyrrolydone). An equal volume of buffer (10 mM TrisHCl, pH 8.0) and saturated phenol were then added to the samples. After 30 min of shaking at 4°C, the phases were separated by centrifugation (10,000 *g*, 30 min, 4°C). The phenolic phase was recovered and extracted with an equal volume of extraction buffer. Proteins were precipitated from the phenolic phase by the addition of 30 mL of 0.1 M ammonium acetate in methanol and incubated for 1 h at −20°C, followed by centrifugation (16,000 *g*, 30 min, 4°C). The pellet was washed three times with ammonium acetate in methanol and once in 30 mL cold acetone (100%), incubated at −20°C for 1 h, followed by centrifugation (16,000 *g*, 30 min, 4°C). The resulting pellet was dried and solubilized in 800 μL of TCT buffer (7 M urea, 2 M thiourea, 10 mM DTT, and 0.1% (v/v) Triton X-100). Complete protein solubilization was achieved by vigorously shaking with vortex. Protein extracts were desalinized by Amicon^®^Ultra-0.5 mL 3K-NMWL filter devices (Millipore).

The protein concentration was determined using the Bradford assay (Bradford, 1976) and confirmed by SDS PAGE electrophoresis (Laemmli, 1970). Fifty micrograms of protein sample were denatured with 25 μL of 0.2% *Rapi*Gest SF (Waters) at 80°C for 15 min; reduced with 2.5 μL of 100 mM dithiothreitol (GE Healthcare) at 60°C for 30 min; and alkylated with 2.5 μL of 300 mM iodoacetamide (GE Healthcare) at RT for 30 min in the dark. Samples were then enzymatically digested with trypsin (sequencing grade modified trypsin, Promega) at a 1:100 (w/w) enzyme: protein ratio. After digestion, 10 μL of 5% trifluoroacetic acid was added to the digested mixture. The digested samples were then incubated at 37°C for 90 min to hydrolyze the *Rapi*Gest. After digestion, the mixture of peptides was desalted using the ZipTip Reversed-Phase ZipTip C18, according to the manufacturer (Millipore). The final volume of 50 μL was obtained by the addition of ammonium formate (20 mM pH 10) solution, containing the internal standard (β*-*phosphorylase at 1 pmol.μL^−1^), to the lyophilized desalted peptide sample.

### MS^E^ Analysis

Mass spectra of the peptide fragments were acquired by reverse-phase ultraperformance liquid chromatography (2D Technology nanoACQUITY-Waters, Corp., Milford, USA). First-dimension separation was achieved in an XBridge BEH130 C18 5 μm 300 μm × 50 mm column (Waters, Corp., Milford, USA). Elution was performed using five different binary gradients with 20 mM pH 10 ammonium formate in acetonitrile at 400 nL/min. Eluted peptides from the first-dimension column were trapped in a Symmetry C18 5 μm 180 μm × 20 mm column (Waters, Corp., Milford, USA) and diluted, online, with acetonitrile containing 0.1% formic acid at a flow rate of 2 μL/min. Second-dimension separation was performed in a HSS T3 1.8 μm 75 μm × 100 mm column (Waters, Corp., Milford, USA) using a binary gradient from 3 to 85% of acetonitrile with 0.1% formic acid for 52 min at a flow rate of 350 ηL/min.

Mass spectrometry acquisition was achieved in a Synapt G2 HDMS mass spectrometer equipped with an ion mobility cell and a nanolockspray source in the positive ion and “V” mode (Waters, Corp., Milford, USA). The MS/MS fragment ions of Glu 1-fibrinopeptide B (GFP) with a doubly charged ion [M + 2H]^2+^ = 7,858,426 *m/z* (Waters, Corp., Milford, USA) was used as lock mass to obtain the final instrument calibration. Data-independent scanning (MSE) experiments were performed by switching between low (3 eV) and elevated collision energies (15–50 eV), applied to the trap “T-wave” cell filled with argon. Scan time of 0.8 s was used for low and high energy scans from *m/z* 50–2,000 (Silva et al., 2006).

### Processing Parameters and Database Search

The raw data processing of protein identification and relative quantitative analyses were all performed using the ProteinLynx Global Server (PLGS 2.5.1). The processing parameters included the following: automatic tolerance for precursor and product ions, minimum of three fragment ions matched per peptide, minimum of seven fragment ions matched per protein, minimum of two peptides matched per protein, one possible trypsin missed cleavage, carbamidomethylation of cysteine as the fixed modification and oxidation of methionine as the variable modification, and a positive false discovery rate (FDR) below 4%. However, we considered only proteins with FDR ≤ 1%. All proteins that do not meet this criterion were excluded.

To identify and quantify the proteins, the intensities of the spectra were calculated by the stoichiometric method, according to the internal standard, by MSE analysis (Silva et al., 2006) and normalized using the PLGS autonormalization function. The amount and sequence of the matched peptide and protein *f*mols were obtained based on the ratio of its three most abundant peptides (“High Top 3” method) determined in each individual experiment (Silva et al., 2006), considering the data from three biological replicates for each sample, described as follows: I_5_-4M, I_9_-4M, I_5_-7M, I_9_-7M and I_5_-10M, I_9_-10M. The FDR for peptide and protein identification was determined based on the search of a reversed database, which was generated automatically using PLGS 2.5.1 by reversing the sequence of each entry. All protein hits were identified with a confidence of >95%. Only those proteins identified in at least two out of the three replicates performed for each biological sample were regarded as having undergone a significant change. The sigmoid distribution (Geromanos et al., 2011), within the dynamic range spectra of the log-transformed data (not shown), was generated by the MassPivot Software (Murad and Rech, 2012), kindly supplied by Murad.

The predicted protein identifications were obtained with the embedded ion accounting algorithm of PLGS Software searching in the database for *Saccharum* spp. (SUCEST) comprising of the transcript sequences from the sugarcane genome project (http://sucest-fun.org/) publicly available (Vettore et al., 2001), to which the sequence of rabbit phosphorylase (Uniprot entry: P00489) was appended as the internal standard to provide the ability to address technical variation and accommodate concentration determinations (Silva et al., 2006). The PLGS 2.5.1 expression data values of *p* < 0.05 and *p* > 0.95 were considered as statistically significant for low or high abundance, respectively, considering the quantitative protein ratio between I_9_ and I_5_.

### Functional Categorization and GO Enrichment Analyses

The matched protein sequences with ionized peptides were functionally categorized. The terms of Gene Ontology Consortium (GO) (Ashburner et al., 2000) (http://www.geneontology.org) were obtained using the Blast_2_GO Software (Conesa et al., 2005), considering the default parameters specified by the program (E-value hit filter 1.0E-6, annotation cut off 55, GO weight 5, HSP-hit coverage cut off 20). The annotations were previously simplified using the feature GoSlim program, and the EC numbers beyond the annotated sequences, with signal peptides (http://www.cbs.dtu.dk/services/SignalP/. dk / services /SignalP/), were obtained by the InterProScan tool (http://www.ebi.ac.uk/interpro/); data not shown.

The GO enrichment analyses relative to the entire proteomic data were performed using the Fisher Exact Test (Ji et al., 2011) with a FDR < 0.05 and *p* < 0.01 in the BLAST_2_GO Software, considering only the most specific GoTerms.

## Results

### Sugar Accumulation in Culms at Different Developmental Stages

The accumulation of soluble sugars in the storage parenchyma, comprising the apoplast and symplast cells in the internodes, was determined from I_4_ to I_9_ at three stages of development (4M, 7M, and 10M). Overall, the concentration of total soluble sugars in the storage parenchyma increased as plants aged (Table 1), reaching its maximum in the mature internode I_9_ at 10M, and after 10 days of drought stress. Internodes I_4_-I_8_, from 4M- to 7M-old-plants, showed the highest content of glucose and fructose, compared to sucrose. However, as the plants matured (10M), sucrose became the predominant stored sugar, particularly in older internodes (I_7_ and I_9_).

**Table 1:** Concentration of glucose, fructose and sucrose in the sugarcane storage parenchyma from 4M, 7M, and 10M-old plants of the sugarcane cultivar SP80-3280, measured by HPLC.

**Internode**	Soluble sugars (g/L)^*^± SE											
	**4-M**				**7-M**				**10-M**			
	**Glucose**	**Fructose**	**Sucrose**	**Total**	**Glucose**	**Fructose**	**Sucrose**	**Total**	**Glucose**	**Fructose**	**Sucrose**	**Total**
4	2.28 ± 0.37	2.38 ± 0.20	4.92 ± 0.16	9.58	9.82 ± 2.85	11.74 ± 2.69	3.81 ± 0.65	25.37	20.31 ± 1.54	21.14 ± 2.53	12.63 ± 4.39	49.48
5	7.04 ± 1.42	7.46 ± 1.53	3.37 ± 1.20	17.88	13.74 ± 1.03	14.67 ± 0.69	4.05 ± 0.83	32.46	24.66 ± 1.76	22.04 ± 2.72	24.48 ± 6.91	71.19
6	9.18 ± 1.64	8.91 ± 1.20	4.22 ± 1.38	22.32	14.92 ± 2.67	16.37 ± 1.40	5.85 ± 0.64	37.14	13.46 ± 1.73	13.61 ± 0.96	44.86 ± 4.93	71.93
7	14.65 ± 3.17	13.25 ± 1.43	4.24 ± 0.38	32.14	12.33 ± 2.05	12.84 ± 1.03	8.38 ± 3.04	37.56	18.76 ± 5.21	16.59 ± 5.14	74.05 ± 2.86	109.40
8	13.47 ± 0.71	11.22 ± 1.26	8.91 ± 2.14	33.61	17.16 ± 1.95	15.83 ± 1.57	13.69 ± 2.72	46.68	13.36 ± 4.51	13.53 ± 4.34	90.08 ± 7.85	116.96
9	8.84 ± 2.14	10.90 ± 3.16	22.64 ± 3.91	42.39	15.09 ± 2.12	16.60 ± 4.35	20.66 ± 1.40	52.34	13.56 ± 0.67	13.37 ± 0.74	107.15 ± 21.43	134.08

* *Values are expressed as mean of 6 biological replicates ± standard error of the mean (SE)*.

*The storage parenchyma comprises the apoplast and symplast cells*.

### Changes in Protein Profile and Abundance in Developing and Maturing Internodes

**Figure 1:**
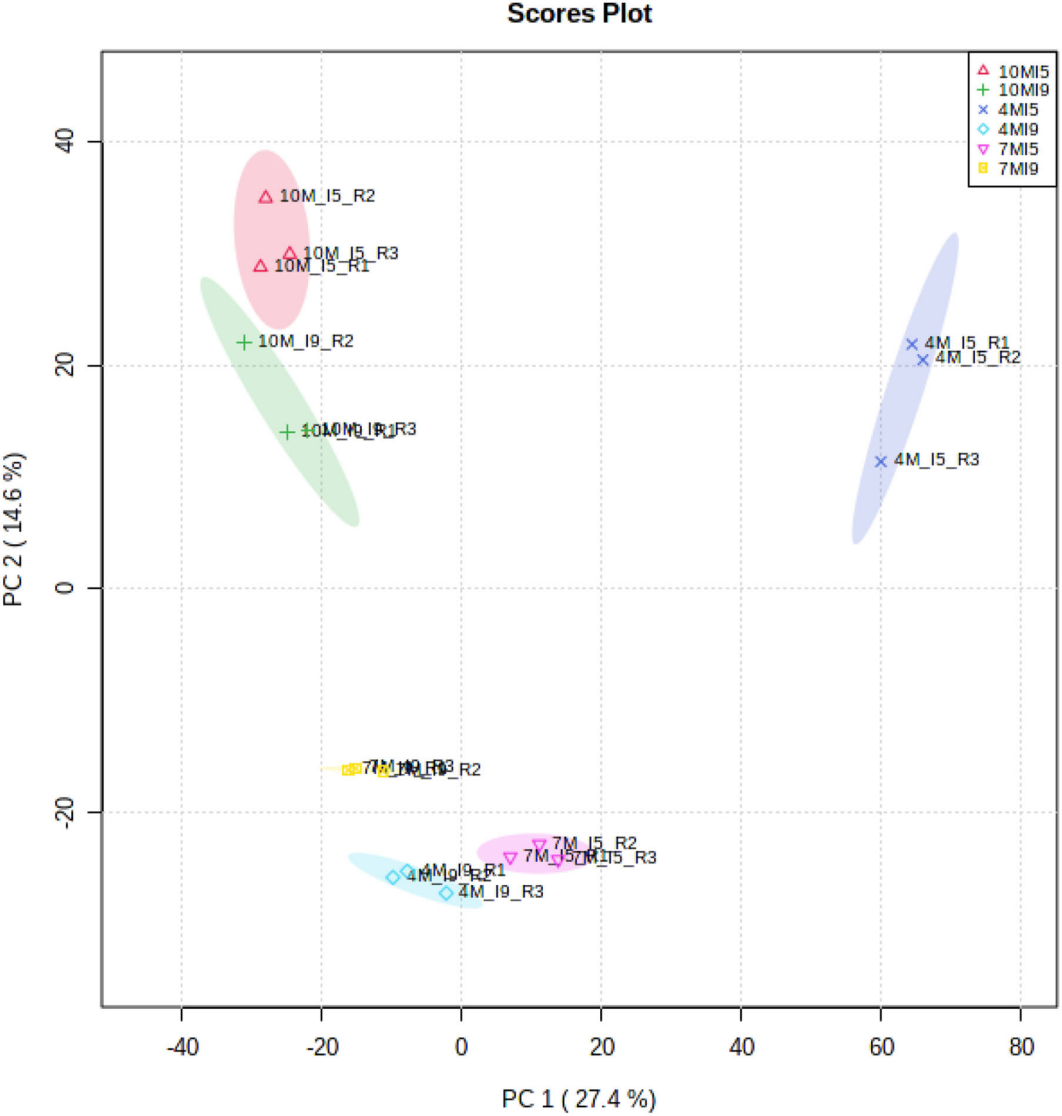
Principal component analysis considering the *f*mol of 1,655 proteins identified by the MassPivot Software, including solely the accession numbers with FDR ≤ 1%, for (I_5_) and (I_9_) analyzed at 4M (I_5_-4M, I_9_-4M), 7M (I_5_-7M, I_9_-7M) and after the drought stress at 10M (I_5_-10M, I_9_-10M).

**Figure 2:**
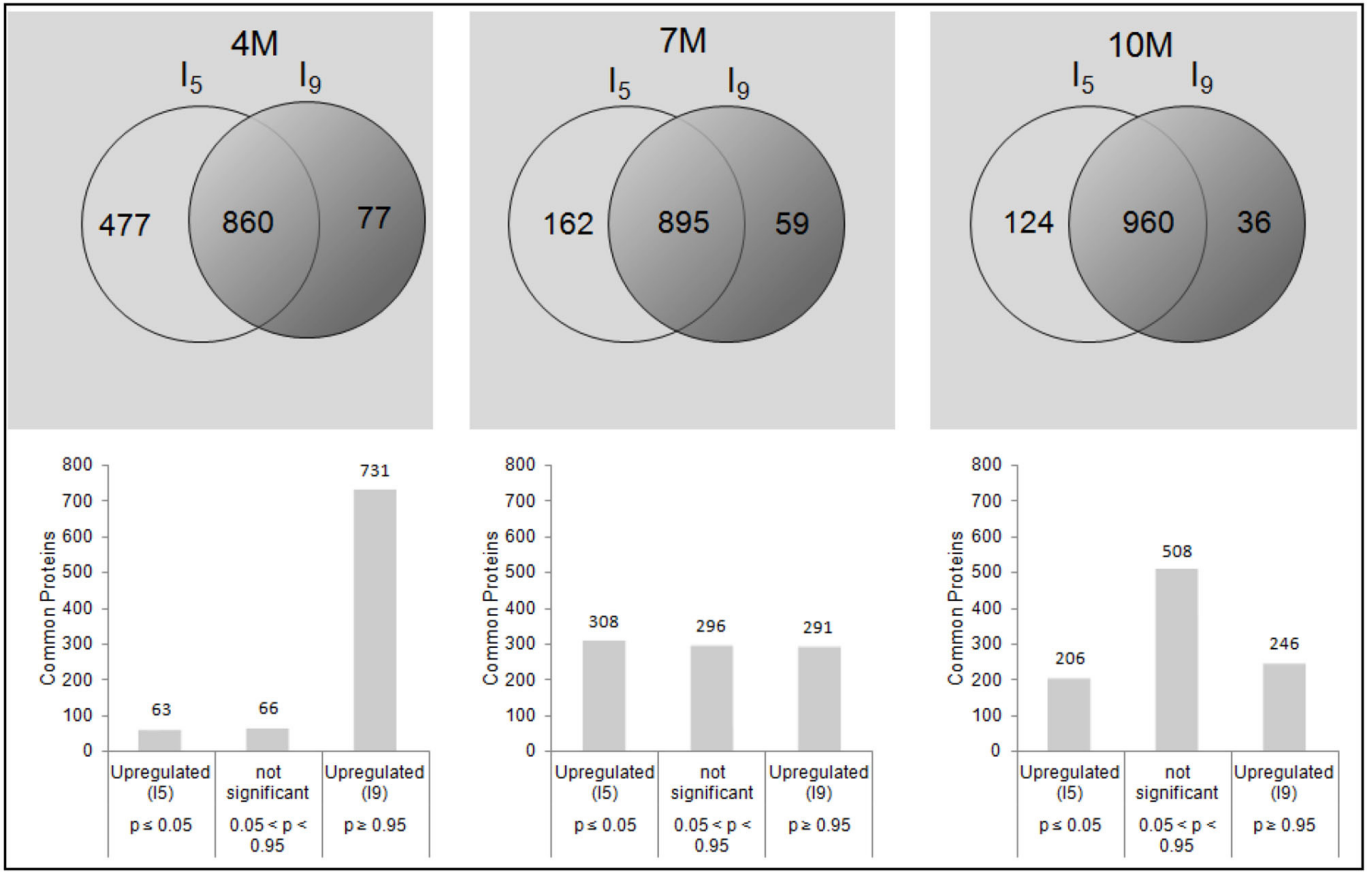
Venn diagrams relative to the total number of common and unique proteins identified by the PLGS 2.5.1 Software, including all different accession numbers with FDR ≤ 1%, for I_5_ and I_9_, analyzed at 4M, 7M, and after the drought stress at 10M. The values of *p* < 0.05 and *p* > 0.95 were considered as statistically significant for low or high abundance, respectively, for the I_9_/I_5_ ratio.

Figure 1 shows the principal component analysis (PCA) for the distribution of all proteins identified and their respective quantities, expressed in *f*mol, for I_5_ and I_9_, at each stage of development (4M, 7M, and 10M) (Supplementary Tables 4–6). Overall, protein profiles and abundance identified in both internodes showed the lowest differences in matured 10M-old-plants. The largest differences were seen between I_9_ and I_5_ at 4M, with 7M showing an intermediate value. Table 2 shows the total number of proteins identified per internode and the average FDR for each biological replicate. Considering the numbers of common proteins, in both the internodes, there was a steady increase, starting with 860 (4M), 895 (7M) up to 960 at 10M, suggesting that as the internodes matured, the metabolism of both I_5_ and I_9_, became similar (Figure 2). This assumption is further corroborated when we compare the unique proteins found in these experimental conditions. In both internodes, the numbers of unique proteins reduced dramatically, from 4M to 10M, particularly for I_5_, which started with 477 (4M), 162 (7M), and reduced to 124 (10M). In the case of I_9_, the number of unique proteins was even lower, starting from 77 in 4M and reducing to 36 in 10M (Figure 2).

**Table 2:** Summary of identified proteins.

	**n° of proteins^a^**				**Protein FDR^b^ (%)**				**n° of common**
**Sample**	**R1**	**R2**	**R3**	**Mean (a)**	**R1**	**R2**	**R3**	**Mean (b)**	**Proteins^c^**
4M-I_5_	1,530	1,528	1,313	1,457	0.10	0.13	0.12	0.12	988
4M-I_9_	866	1,020	868	918	0.01	0.05	0.03	0.03	615
7M-I_5_	1,009	1,248	1,236	1,164	0.03	0.05	0.06	0.05	780
7M-I_9_	905	1,086	988	993	0.04	0.09	0.05	0.06	654
10M-I_5_	1,040	1,333	1,235	1,203	0.01	0.04	0.06	0.04	800
10M-I_9_	973	1,087	1,077	1,046	0.02	0.09	0.04	0.05	708

a *Number of proteins identified for each biological replicate (R1, R2, and R3) and their respective mean*.

b *FDR (%) = False discovery rate for each replicate and their respective mean in percentage*.

c *Number of common proteins to all 3 replicates*.

We then used the ratio I_9_/I_5_ for the abundance analyses of common proteins at each stage of development, to identify proteins with higher or lower content in internode 9, compared to internode 5 of the same plant (Supplementary Tables 1–3). In 4M old-plants, 63 proteins showed a significant increase in abundance in I_5_, whereas 731 proteins were more abundant in I_9_. In 7M-old-plants 308 proteins showed higher abundance in I_5_, whereas it was 291 in I_9_ (Figure 2). In 10M plants 206 were significantly more abundant in I_5_, whereas 246 were significantly more abundant in I_9_ (Figure 2).

**Figure 3:**
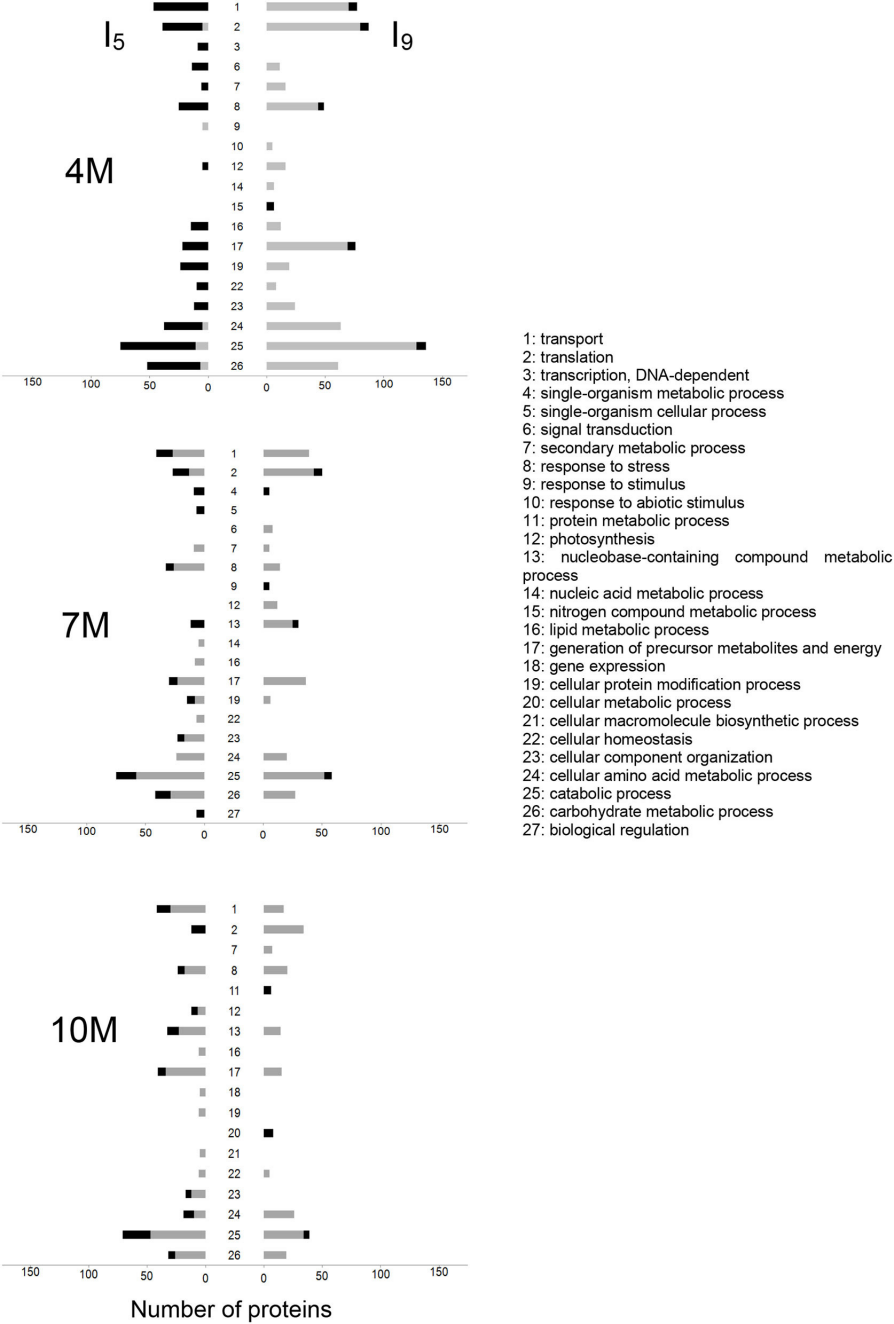
Distribution of differential abundance of the proteins, identified with PLGS 2.5.1 Software, for I_5_ (left) and I_9_ (right), at 4 M, 7M, and 10M. Proteins were classified according to the BLAST_2_GO annotation for biological processes. Black and gray bars represent the unique and highly abundant sequences, respectively. The GO enrichment analysis, relative to the entire proteomic data, was performed using the Fisher Exact Test with FDR < 0.05 and *p* < 0.01 in the BLAST_2_GO Software, considering only the most specific GOTerms.

Following the identification, we then classified the proteins into three functional categories using BLAST_2_GO as follows: molecular function, biological process, and cellular components. Enrichment analysis, according to the Fisher's Exact Test (Ji et al., 2011), reduced to the most specific GoTerm, identified statistically significant differences between I_5_ and I_9_, from 4M-, 7M-, and 10M-old-plants (Supplementary Figure 1). The number of functional categories increased as the plants matured from two categories at 4M to nine in 10M internodes (Supplementary Figure 1). Figure 3 shows the distribution of differentially abundant proteins classified according to the biological process they were involved. Proteins were identified by the PLGS Software for both internodes (I_5_ and I_9_) at each stage of development (4M, 7M, and 10M), and classified according to the BLAST_2_GO annotation. In internode I_9_ of 4M, the categories of catabolic process, translation, transport, and preparation of precursor metabolites and energy had the highest number of proteins with high abundance. In 4M-old-plants the number of unique proteins present in I_5_ was particularly higher than the values observed for both internodes at 7 and 10M. Throughout the development of the plant, some categories were dramatically reduced in content, reaching its minimum values at I_9_-10M such as transport, generation of precursor metabolites and energy, photosynthesis, secondary metabolic process, catabolic process, and carbohydrate metabolic process. These changes in protein abundance between I_9_ and I_5_ provided a clear view that major changes in the proteome was taking place as sucrose accumulated in maturing internodes (Figure 3).

### Quantitative Changes of Proteins Involved in Carbon Metabolism and Sucrose Accumulation

The accumulation of sucrose in the parenchymatic tissues is dependent on a series of metabolic pathways, such as glycolysis and respiration, which will regulate its fate upon transport from leaves. Distinct invertases located in the cell wall, vacuoles, and cytosol regulate the breakdown of sucrose directing its constituent reducing hexoses, either to glycolysis and respiration, cell wall polysaccharides, or sucrose resynthesis for storage in the vacuoles (Huang et al., 2007). Table 3 shows the identification of two proteins of soluble acid invertase. The first was unique to I_5_-4M and 7M, and the second was unique to I_5_-7M. We also found a non-specific sucrose cleavage enzyme, statistically significant and more abundant in 4M, which could be an acid alkaline invertase or even a SuSy.

**Table 3:** Fold changes (log_e_I_9_/I_5_ ratio) at (4M, 7M, and 10 M) in the abundance of specific common proteins with FDR ≤ 1% identified by LC-MS^E^.

**Accession**	**Protein description**	**Enzyme code**	**4M-I_**9**_/I_**5**_**	**7M-I_**9**_/I_**5**_**	**10M-I_**9**_/I_**5**_**
	**Sucrose cleavage and accumulation**				
SCVPLR2012D11	Non-specific sucrose cleavage enzyme	–	2.0	–	–
SCCCST3006A04	Soluble acid invertase (ACIN)	EC:3.2.1.26	4MI5	7MI5	–
SCCCLR1C07F04	Soluble acid invertase (ACIN)	EC:3.2.1.26	–	7MI5	–
SCVPRZ2040D09	Invertase inhibitor	EC:3.1.1.11	4MI9	–	1.8
	**Glycolysis**				
SCCCCL4011H08	Pyrophosphate fructose 6 phosphate 1 phosphotransferase (PFP)	EC:2.7.1.90	0.7	0.6	–
SCCCFL4092E06	Pyrophosphate fructose 6 phosphate 1 phosphotransferase (PFP)	EC:2.7.1.90	–	0.6	–
SCCCCL3001B07.b	Pyrophosphate fructose 6 phosphate 1 phosphotransferase (PFP)	EC:2.7.1.90	1.9	–	1.3
SCCCCL3120B01	Fructokinase	EC:2.7.1.4	3.3	0.6	–
SCJLRZ1021E10	Fructokinase	EC:2.7.1.4	3.4	0.6	1.2
SCRFLR1012E06	Fructokinase	EC:2.7.1.4	3.2	–	–
SCRULR1020D11	Fructose bisphosphate aldolase	EC:4.1.2.13	1.2	0.6	0.6
SCCCCL3120C03	Fructose bisphosphate aldolase	EC:4.1.2.13	3.3	1.5	1.2
SCCCCL3005E06.b	Fructose bisphosphate aldolase	EC:4.1.2.13	0.5	0.7	0.8
SCCCRZ2001F03	Fructose bisphosphate aldolase	EC:4.1.2.13	5.0	1.9	1.3
SCACSB1037G08	Fructose bisphosphate aldolase	EC:4.1.2.13	4MI5	–	1.2
SCQSLR1061E07	Glucose 6 phosphate isomerase (PGI)	EC:5.3.1.9	2.5	1.6	1.3
SCEQRT1029D09	Glucose 6 phosphate isomerase (PGI)	EC:5.3.1.9	1.3	–	–
SCCCRZ1001B08	Triosephosphate isomerase cytosolic	EC:5.3.1.1	2.3	0.8	0.91
SCEPRZ1009B12	Triosephosphate isomerase cytosolic	EC:5.3.1.1	0.6	1.3	1.48
SCCCCL2001B02.b	NAD-dependent glyceraldehyde 3 phosphate dehydrogenase (GAPDH)	EC:1.2.1.12	0.9	1.5	–
SCCCCL3001G02	NAD-dependent glyceraldehyde 3 phosphate dehydrogenase (GAPDH)	EC:1.2.1.12	2.7	1.1	0.9
SCCCLR1001E06	NAD-dependent glyceraldehyde 3 phosphate dehydrogenase (GAPDH)	EC:1.2.1.12	1.8	0.8	0.8
SCCCLR1072B04	NAD-dependent glyceraldehyde 3 phosphate dehydrogenase (GAPDH)	EC:1.2.1.12	4.0	1.7	–
SCBGLR1099C04	NAD-dependent glyceraldehyde 3 phosphate dehydrogenase (GAPDH)	EC:1.2.1.12	8.8	–	–
SCUTSD2028F03	NAD-dependent glyceraldehyde 3 phosphate dehydrogenase (GAPDH)	EC:1.2.1.12	–	–	0.9
SCSBSB1052D07	NAD-P-dependent glyceraldehyde 3 phosphate dehydrogenase	EC:1.2.1.13	5.7	1.3	–
SCVPRT2083B12	Cytosolic phosphoglycerate kinase 1 (PGK)	EC:2.7.2.3	4MI9	0.8	0.7
SCCCLR1079B06	Cytosolic phosphoglycerate kinase 1 (PGK)	EC:2.7.2.3	3.1	1.7	–
SCEZLB1006F11	Phosphoglycerate kinase (PGK)	EC:2.7.2.3	3.1	1.2	–
SCCCRZ1001C05	Phosphoglycerate kinase (PGK)	EC:2.7.2.3	2.9	1.3	–
SCCCRZ1002F06	Enolase	EC: 4.2.1.11	2.6	0.9	0.8
SCJFLR1073H09	Enolase	EC: 4.2.1.11	3.7	–	1.1
SCEZLB1006G09	Pyruvate kinase cytosolic isozyme	EC:2.7.1.40	5.8	2.0	0.9
SCCCRZ1002E05	Pyruvate kinase cytosolic isozyme	EC:2.7.1.40	3.4	–	1.4
SCJFRZ2029D10	Pyruvate kinase cytosolic isozyme	EC:2.7.1.40	2.5	–	1.6
SCCCLR1070D04	Pyruvate kinase cytosolic isozyme	EC:2.7.1.40	1.3	1.4	0.9
	**Oxidative Pentose Phosphate Pathway**				
SCUTLR1037G10	Glucose 6 phosphate dehydrogenase	EC:1.1.1.49	–	0.7	1.3
SCMCST1049F11	NAD-P-dependent 6 phosphogluconate dehydrogenase	EC:1.1.1.44	4.3	1.3	–
SCQSLR1040E09	NAD-P-dependent 6 phosphogluconate dehydrogenase	EC:1.1.1.44	6.4	1.4	–
SCBFAD1067A05	NAD-P-dependent 6 phosphogluconate dehydrogenase	EC:1.1.1.44	–	–	2.0
	**Sugar Nucleotide Biosynthesis**				
SCACSB1117F03	Sucrose synthase (SUSY)	EC:2.4.1.13	0.4	0.3	–
SCCCLR1001A05	Sucrose synthase (SUSY)	EC:2.4.1.13	1.6	1.1	0.8
SCVPST1061C06	Sucrose synthase (SUSY)	EC:2.4.1.13	2.6	–	–
SCEZLR1052C03	Sucrose synthase (SUSY)	EC:2.4.1.13	2.0	1.4	0.7
SCCCRZ1002G07	Sucrose synthase (SUSY)	EC:2.4.1.13	0.5	0.1	–
SCEZRZ3099D06	Sucrose synthase (SUSY)	EC:2.4.1.13	3.5	–	–
SCQGLR1019G02	UDP glucose 6 dehydrogenase	EC:1.1.1.22	1.3	0.3	0.6
SCJFLR1013H10	UDP glucose 6 dehydrogenase	EC:1.1.1.22	0.9	0.3	0.6
SCQGLR1062D04	UDP glucose pyrophosphorylase	EC:2.7.7.9	4.0	1.2	–
	**Ethanolic Fermentation and Acetate Metabolism**				
SCJFRT1062H07	Pyruvate decarboxylase	EC:4.1.1.1	4MI9	–	0.8
SCCCCL4011D12	Pyruvate decarboxylase	EC:4.1.1.1	2.0	1.1	–
SCJFRT1005D04	Pyruvate decarboxylase	EC:4.1.1.1	1.8	1.2	–
SCCCLR2001H05	Alcohol dehydrogenase 1	EC:1.1.1.1	5.0	–	0.9
SCCCST2003C12	Alcohol dehydrogenase 1	EC:1.1.1.1	3.1	1.2	0.8
SCCCCL4006H08	Alcohol dehydrogenase 2	EC:1.1.1.1	24.0	1.1	–
SCCCLR1066H09	Alcohol dehydrogenase class 3	EC:1.1.1.1	1.4	1.1	1.2
SCCCRZ2001F10	Mitochondrial NAD-aldehyde dehydrogenase	EC:1.2.1.3	2.3	–	–
SCJFLR1073D12	Mitochondrial NAD-aldehyde dehydrogenase	EC:1.2.1.3	8.4	–	0.8
SCACAD1036F01	Mitochondrial NAD-aldehyde dehydrogenase	EC:1.2.1.3	4.3	–	0.7
SCJLRZ1021D03	Acetyl-CoA synthetase	EC:6.2.1.1	1.4	–	–
SCCCCL3002A09.b	Alanine aminotransferase	EC: 2.6.1.2	2.5	–	0.8
	**Pyruvate and Malate Biosynthesis**				
SCJLRZ1024A01	Phosphoenolpyruvate carboxylase	EC:4.1.1.31	2.3	–	–
SCCCCL3001F10.b	Phosphoenolpyruvate carboxylase	EC:4.1.1.31	4.2	1.5	–
SCCCLR1072A12	Malate dehydrogenase cytoplasmic	EC:1.1.1.37	1.7	0.6	–
SCEPRZ3084H02	Malate dehydrogenase cytoplasmic	EC:1.1.1.37	1.1	1.3	0.6
SCCCCL4004C11	Malate dehydrogenase cytoplasmic	EC:1.1.1.37	1.1	0.6	0.7
SCCCLR1067B04	Malate dehydrogenase cytoplasmic	EC:1.1.1.37	1.1	0.6	0.7
SCCCLR1024D03	Malate dehydrogenase cytoplasmic	EC:1.1.1.37	–	–	0.8
	**Tricaboxylic Acid Cycle-TCA**				
SCCCCL1002D10.b	Pyruvate dehydrogenase	EC:1.2.4.1	2.6	–	–
SCEZLR1009F06	Pyruvate dehydrogenase	EC:1.2.4.1	–	–	1.2
SCJLRT1023A07	Pyruvate dehydrogenase	EC:1.2.4.1	–	–	1.3
SCVPLR2012F11	ATP citrate synthase	EC:2.3.3.8	1.9	–	1.2
SCCCLR1C06C11	ATP citrate synthase beta chain	EC:2.3.3.8	2.8	0.9	–
SCCCCL3001C03	Citrate synthase mitochondrial	EC:2.3.3.8	2.2	–	–
SCCCCL3001B09.b	Aconitate hydratase 1	EC:4.2.1.3	3.4	–	–
SCCCCL3001A05	Aconitate hydratase cytoplasmic	EC:4.2.1.3	2.0	0.8	0.9
SCJFRZ1005G06	Aconitate hydratase cytoplasmic	EC:4.2.1.3	2.0	–	–
SCCCLR1065F02	Aconitate hydratase cytoplasmic	EC:4.2.1.3	1.8	–	0.9
SCJFRZ3C04G04.b	Aconitate hydratase cytoplasmic	EC:4.2.1.3	1.7	–	–
SCCCLR1C02B02	Aconitate hydratase cytoplasmic	EC:4.2.1.3	1.4	–	1.3
SCRLCL6031B10	NAD-dependent isocitrate dehydrogenase	EC:1.1.1.41	2.0	1.2	–
SCCCLR1C04G09	NAD-P-specific isocitrate dehydrogenase	EC:1.1.1.42	1.8	–	–
SCCCCL4017A09	NAD-P-specific isocitrate dehydrogenase	EC:1.1.1.42	1.8	–	–
SCCCCL7C03F06	NAD-P-specific isocitrate dehydrogenase	EC:1.1.1.42	–	–	1.6
	**Respiratory Chain**				
SCSFLR2031H06	Cytochrome C oxidase subunit vb precursor	EC:1.9.3.1	13.60	0.22	1.45
SCQSLB1049A06	Cytochrome C oxidase subunit vb precursor	EC:1.9.3.1	2.59	0.78	0.78
	**Ascorbate/Glutatione Cycle—ROS Scavenging Enzymes**				
SCSGRT2066C02	APX1 cytosolic ascorbate peroxidase	EC:1.11.1.11	3.4	0.6	0.9
SCRURT3064F04	APX1 cytosolic ascorbate peroxidase	EC:1.11.1.11	3.4	0.7	–
SCEQRT1025E05	APX2 cytosolic ascorbate peroxidase	EC:1.11.1.11	3.6	0.5	–
SCAGLR1043C02	APX2 cytosolic ascorbate peroxidase	EC:1.11.1.11	1.6	0.9	–
SCEQRT2099G01	Ascorbate peroxidase chloroplastidial isoform 1	EC:1.11.1.11	3.7	–	–
SCQGLR2010E11	Putative ascorbate peroxidase chloroplastidial	EC:1.11.1.11	3.0	–	2.4
SCCCLR1079D10	Monodehydroascorbate reductase	EC:1.6.5.4	1.7	1.6	–
SCQSRT2031F03	Monodehydroascorbate reductase	EC:1.6.5.4	1.2	7MI9	–
SCCCLR1078G10	Glutathione reductase cytosolic	EC:1.8.1.7	4MI9	–	1.5
SCCCRT1003F01	Glutathione reductase cytosolic	EC:1.8.1.7	3.1	–	1.3
SCCCRZ2C01F01	Glutathione peroxidase	EC:1.11.1.9	6.7	0.7	–
SCVPLR2012H12	Glutathione S transferase	EC:2.5.1.18	3.6	–	0.8
SCJLST1027D09	Glutathione S transferase	EC:2.5.1.18	2.5	–	–
SCQSLB1049C04	Glutathione S transferase 6	EC:2.5.1.18	2.7	-	0.8
SCCCCL3002C09.b	Glutathione S transferase GSTF2	EC:2.5.1.18	8.0	1.1	1.1
SCRULR1042G12	Glutathione S transferase GSTF2	EC:2.5.1.18	3.0	–	–
SCEPAM1018F06	Glutathione S transferase GSTF2	EC:2.5.1.18	0.9	–	0.6
SCJLRZ1027G11	Glutathione transferase	EC:2.5.1.18	20.7	1.2	2.4
SCCCLR1001A07	Glutathione transferase III	EC:2.5.1.18	3.0	1.1	0.8
SCCCCL4003D01	Glutathione transferase 30	EC:2.5.1.18	4MI9	3.9	1.2
SCQSLB1049C07	Glutathione S transferase GSTF2	EC:2.5.1.18	–	0.9	0.6
SCJLRT1022G04	Glutathione S transferase zeta class	EC:2.5.1.18	–	1.8	1.5
SCBGLR1003E05	Glutathione S transferase	EC:2.5.1.18	–	–	0.6
SCSFCL6068E03	Glutathione S transferase	EC:2.5.1.18	–	–	2.4
SCCCLR1048D04	Glutathione S transferase	EC:2.5.1.18	–	–	2.0
SCEZRT3070A09	Glutathione transferase III	EC:2.5.1.18	–	–	0.8
SCMCRZ3064B09	Hemoglobin 2	-	22.4	7MI9	-
SCRURT2006C10	Isoflavone reductase	EC: 1.3.1.45	4MI9	-	-
SCSBST3096H07	Isoflavone reductase	EC: 1.3.1.45	13.1	2.0	1.2
SCVPLB1020E05	Isoflavone reductase	EC: 1.3.1.45	12.7	2.0	-
SCCCLR1048A06	Superoxide dismutase	EC: 1.15.1.1	2.0	1.3	1.2
SCCCLR2C03D05	Superoxide dismutase	EC: 1.15.1.1	6.4	1.3	-
	**Lignin Biosynthesis**				
SCCCRZ2C01F05	Cinnamyl alcohol dehydrogenase 2	EC:1.1.1.195	3.1	–	0.6
SCEQLR1029E05	Putative cinnamyl alcohol dehydrogenase 8c	EC:1.1.1.195	13.9	2.2	1.1
SCEPRZ1011A02	Cinnamyl alcohol dehydrogenase	EC:1.1.1.195	–	0.3	–
SCCCLR1048D07	Phenylalanine ammonia lyase	EC:4.3.1.24	2.2	0.5	1.4
SCJLRT1013C01	Phenylalanine ammonia lyase	EC:4.3.1.24	0.5	0.6	1.4
SCJFLR1017B11	Phenylalanine ammonia lyase	EC:4.3.1.24	–	0.6	1.4
SCVPRZ2035F03	Peroxidase 2	EC: 1.11.1.7	26.3	1.5	0.3
SCCCLB1004B09	Peroxidase-39 precursor	EC: 1.11.1.7	5.7	0.8	0.5
SCVPLB1016F07	Polyphenol oxidase	EC: 1.10.3.1	3.7	1.8	1.1
SCEZLB1007D07	Polyphenol oxidase	EC: 1.10.3.1	2.8	1.9	1.2
SCBFLR1039A03	Polyphenol oxidase	EC: 1.10.3.1	2.2	1.4	-
	**Photosynthesis**				
SCCCLR2C02D12	Chlorophyll a b binding apoprotein CP 26 precursor	–	3.5	1.6	–
SCUTST3086G11	Chlorophyll a b binding protein	–	12.8	1.8	0.8
SCJLST1022G06	Chlorophyll a b binding protein	–	5.2	1.5	10MI5
SCCCST1001B11	Chlorophyll a b binding protein 1	–	7.3	1.5	0.7
SCJFLR1074A11	Chlorophyll a b binding protein 2	–	7.5	1.6	0.7
SCJFSB1014F03.b	Chlorophyll a b binding protein CP 24	–	2.6	1.3	–
SCUTST3131A12	Chlorophyll a b binding protein	–	–	1.6	–
SCACLR2007B05	Chlorophyll a b binding protein CP 24	–	–	1.4	–
SCCCLR1001E04	Chloroplastidial ribulose bisphosphate carboxylase oxygenase small subunit	EC:4.1.1.39	8.8	–	0.7
SCJFLR1073E09	Ribulose bisphosphate carboxylase oxygenase large subunit	EC:4.1.1.39	3.9	1.7	0.8
	**Hormone biosynthesis**				
SCSFRT2068D12	Abscisic acid stress ripening related protein 2	–	4.6	0.7	–
SCCCRT2004B11	Abscisic acid stress ripening related protein 3	–	2.1	–	–
SCCCLR2001A06	Auxin induced protein PCNT115	–	6.0	1.5	1.2
SCVPRT2074E05	Auxin induced protein PCNT115	–	2.9	–	–
SCCCFL1003H07	Auxin induced protein PCNT115	–	1.4	1.4	–
SCJFRZ2007H09	Glutamate decarboxylase	EC:4.1.1.15	3.2	0.6	1.2
SCCCRZ2C03D05	Glutamate decarboxylase	EC:4.1.1.15	1.3	–	1.1

*The values of p < 0.05 and p > 0.95 were considered as statistically significant for low or high abundance, respectively, for the (log_e_I_9_/I_5_ ratio). 4MI5, 4MI9, 7MI5, 7MI9, 10MI5, and 10MI9 represent unique proteins identified for each developmental stage*.

Other important factors involved in the accumulation of sucrose in culms are the invertase inhibitors. We found one invertase inhibitor unique to I_9_-4M and significantly more abundant in I_9_-10M (Table 3). These results provide further evidence that the accumulation of sucrose in maturing sugarcane internodes might be also due to an increase in the expression of genes coding for invertase inhibitors, particularly in 10M-old-plants, as the maturation reaches its maximum.

Upon hydrolysis of sucrose by invertases, the reducing hexoses produced may follow different metabolic pathways whose predominant direction will be determined by the developmental stage of the plant (Patrick et al., 2013). Two enzymes, namely phosphofructokinase (PFK) and pyrophosphate-fructose 6-phosphate 1-phosphotransferase (PFP), play an important role in the regulation of these metabolic pathways. We found two PFKs unique to the juvenile internode I_5_ at 4M (Supplementary Table 4). Two PFP proteins were either absent or repressed in I_9_ in all stages of development, but one was significantly more abundant in I_9_ at 4M and 10M.

Following sucrose hydrolysis, the enzymes hexokinase, fructokinase, fructose bisphosphate aldolase (FBPase), glucose 6-phosphate isomerase (PGI), and triose phosphate isomerase, involved in the first steps of the glycolytic and oxidative pentose phosphate pathways were identified (Table 3). Three proteins of fructokinase were identified, and all were highly abundant in the internode I_9_ of 4M and repressed in the same internode at 7M and 10M. Only one fructokinase was slightly more abundant in I_9_-10M. In the case of fructose bisphosphate aldolase five proteins were identified, showing a predominant increase in abundance in I_9_-4M, with exception of SCCCCL3005E06b, which was repressed at all stages of development. Overall, the enzymes FBPase had their content reduced in I_9_ at 7M and 10M (Table 3). Similar results were observed for PGI, where the two enzymes identified had their maximum abundance in internode I_9_ of 4M, followed by a steady reduction or even absence in the same internode at 7 and 10M.

Two proteins of triose phosphate isomerase were identified having a distinct behavior. One protein was significantly more abundant in I_9_ at 4M, whereas the second was reduced in I_9_-4M, steadily increasing its abundance as the plant aged.

Glyceraldehyde-3-phosphate dehydrogenase (GAPDH) is an important phosphorylating enzyme of the glycolytic pathway in plants, as well as its cytoplasmic non-phosphorylating form that oxidizes G3P (glyceraldehyde 3 phosphate) directly to 3-phosphoglycerate. One non-phosphorylating GAPDH (NADP-dependent glyceraldehyde 3 phosphate dehydrogenase) identified was 5.7-fold-change more abundant in I_9_ of juvenile plants, particularly in 4M, and absent in I_9_-10M. In the case of NAD-dependent glyceraldehyde 3 phosphate dehydrogenase, six proteins were identified showing different levels of abundance, with three being 2.7, 4.0, and 8.8-fold-change higher in I_9_ of 4M. As the plants aged, there was a reduction in abundance of this enzyme in I_9_.

Phosphoglycerate kinase (PGK) and enolase catalyze the reversible reactions of glycolysis. Four proteins of PGK were identified, with three being on average 3.0-fold-change higher in I_9_-4M, reducing in 7M, and being undetected in 10M. One PGK, however, was unique to I_9_-4M and repressed in 7M and 10M. Similar results were observed for two proteins identified as enolases. The last enzyme of the glycolytic pathway, responsible for the phosphorylation of phosphoenolpyruvate (PEP) and conversion to pyruvate, is pyruvate kinase (PK). We found four PKs and all were highly abundant (1.3- to 5.8-fold-change) in I_9_-4M, reducing its content in the later stages of development (10M), with two undetected in 7M.

Glucose 6 phosphate dehydrogenase (G6P-DH) directs the flux of glucose toward the formation of pentose phosphates. Table 3 shows one protein highly abundant in I_9_-10M, reduced in I_9_-7M, and not detected in the same internode at 4M. The other enzyme, involved in the oxidative pentose phosphate pathway identified, was the NADP-dependent 6-phosphogluconate dehydrogenase. Table 3 shows four proteins identified, with two being highly abundant in I_9_-4M by 4.3 and 6.4-fold-change, reducing its abundance in 7M and not being present in I_9_-10M. The other two proteins increased their content, reaching the maximum values of 1.3 and 2.0-fold-change in 10M.

Sucrose synthase, UDP-glucose pyrophosphorylase, and UDP-glucose 6 dehydrogenase are important enzymes regulating the sugar nucleotide biosynthesis, which will be incorporated into the cell wall as cellulose and hemicelluloses. SuSy starts the pathway hydrolyzing sucrose to supply hexoses for the glycolytic and pentose phosphate pathway and/or for cell wall biosynthesis. Six SuSy proteins were identified, having different abundance levels during all stages of development (Table 3). Four showed significant higher abundance in I_9_ of 4M-old plants, varying from 1.6- to 3.5-fold-changes. All these proteins reduced their abundance as the plants aged, becoming repressed in 10M. The other two proteins identified were repressed or not identified in I_9_.

Table 3 shows the identification of two UDP-glucose 6 dehydrogenases, with accession SCQGLR1019G02 being slightly higher in abundance at 4M and repressed in 7 and 10M, whereas accession SCJFLR1013H10 was repressed in I_9_ at all stages of plant development. We identified a UDP-glucose pyrophosphorylase abundant in I_9_ of 4M and 7M, particularly at 4M, which showed a 4.0-fold-change.

### Identification of Proteins Involved in Ethanolic Fermentation and TCA

Ethanol fermentation is a metabolic route used by plants to survive under anaerobic conditions, such as flooding (Geigenberger, 2003; Bailey-Serres and Voesenek, 2008, 2010). In such circumstances, mitochondrial respiration is impaired by low oxygen availability, reducing ATP production in the respiratory chain; the increases in glycolytic and TCA flux is the response of the plant to overcome the limitation of ATP production (Tadege et al., 1999; Bailey-Serres and Voesenek, 2008, 2010; Shingaki-Wells et al., 2014). The enzyme pyruvate decarboxylase (PDC) is responsible for the decarboxylation of pyruvate into acetaldehyde, which is subsequently converted to ethanol by alcohol dehydrogenase (ADH), regenerating NAD^+^ which will be used in glycolysis and the TCA cycle. We identified three PDCs which were either unique or highly abundant in I_9_-4M, reducing their content as the plant aged (Table 3). Similarly, we found four ADH proteins, with their respective proteoforms 1, 2, and 3. In all cases, these proteins were highly abundant in I_9_-4M, with ADH-2 reaching a 24-fold-change. As the plants continued to develop, the abundance of all ADH enzymes were reduced, reaching the lowest values at 10M. These results suggest that the parenchymatic tissues in internode 9 of 4M-old-plants are under hypoxia, probably due to the long distance from the shoot apical meristem and leaves, and by the barrier to gas diffusion imposed by the waxed bark (see Supplementary Figure 2). Besides pyruvate, the ethanolic fermentation can also produce alanine as an end-product (Bailey-Serres and Voesenek, 2008). In fact, we found one alanine aminotransferase significantly induced (2.5-fold-change) in I_9_-4M, which was absent in I_9_ at 7M and 10M (Table 3). The metabolite analysis identified alanine in both internodes (I_5_-4M and I_9_-4M), but with no major differences in content (see Supplementary Table 7). On the other hand, asparagine accumulated in I_9_-4M (see Supplementary Table 8). This amino acid is known to act both as a source of amide/amino nitrogen and as a nitrogen storage compound in plants (Gaufichon et al., 2016); the accumulation of asparagine in I_9_-4M might indicate nitrogen storage during culms maturation.

**Figure 4:**
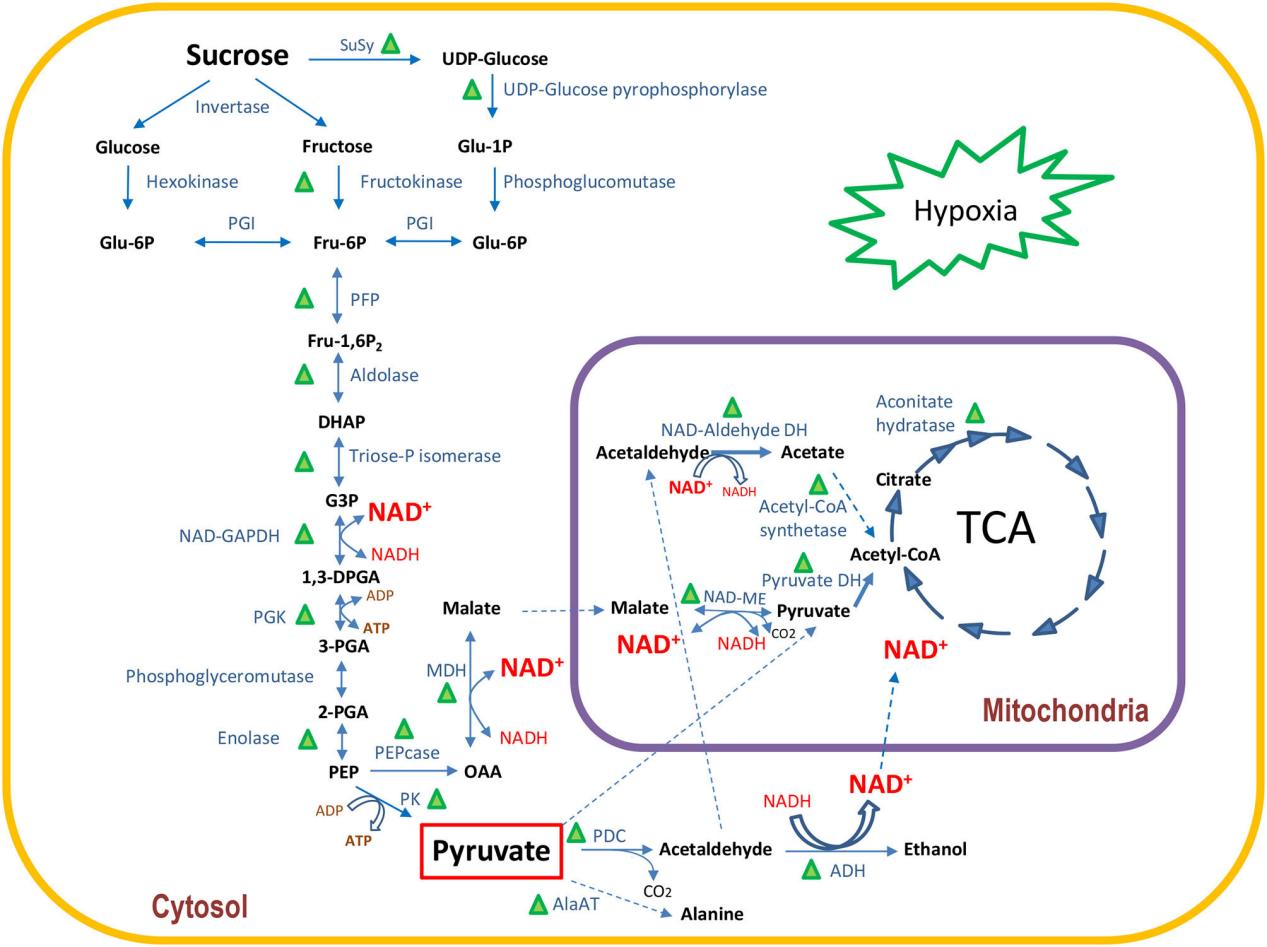
Schematic view of the quantitative changes in enzymes involved in primary carbon metabolism under hypoxia in I_9_ relative to I_5_ in 4-month-old-plants (data from Table 3). Green triangles indicate enzymes with at least one proteoform with a statistically significant increase in abundance.

The acetaldehyde produced by PDC is toxic to plant cells and needs to be converted into another compound to avoid cell damage. One possibility is the conversion to acetyl-CoA in the cytoplasm by acetaldehyde dehydrogenase. Although we did not find any cytoplasmic acetaldehyde dehydrogenase, we identified a mitochondrial NAD-aldehyde-DH which can convert acetaldehyde to acetate (Table 3 and Figure 4). Acetate can be further metabolized to acetyl-CoA by acetyl-CoA synthetase and, therefore, entering the TCA cycle. In fact, we identified three mitochondrial NAD-aldehyde dehydrogenases and one acetyl-CoA synthetase, which were all highly abundant in I_9_-4M and highly reduced or not even found in matured internodes (Table 3).

Malate is formed in the cytosol by the carboxylation reaction of phosphoenolpyruvate (PEP), catalyzed by phosphoenolpyruvate carboxylase (PEPcase). The reaction forms the intermediate metabolite, oxaloacetate (OAA), which is immediately converted to malate by malate dehydrogenase (MDH). Five NAD-dependent MDH were identified, all showing a significant increase in abundance in internode 9 of 4M, reducing their abundance in I_9_ as the plant aged, with the exception of one protein, which maintained its content in 7M. Interestingly, we identified one chloroplast NADP-dependent MDH which was highly abundant in I_9_-4M, showing a 12.4-fold change. This enzyme was detected as unique to I_9_ in 7M plants, and absent in the same internode at 10M.

The mitochondrial pyruvate dehydrogenase complex catalyzes the conversion of pyruvate into acetyl-CoA, which is one of the entry sites for the TCA cycle. We found three proteins of pyruvate dehydrogenase, one highly abundant in I_9_-4M and not present in the same internode at 7M and 10M. The other two proteins were mainly abundant in the late stage of development in I_9_ (10M), and not found in the same internode of 4M and 7M (Table 3). The other enzymes involved in the first steps of TCA cycle are citrate synthase, aconitate hydratase, and isocitrate dehydrogenase. Three proteins representing citrate synthase were identified, one mitochondrial aconitate hydratase and five cytoplasmic and four of isocitrate dehydrogenase. All the proteins were significantly more abundant in I_9_-4M, reducing their content in 7M and 10M. The exception was one isocitrate dehydrogenase, which was not identified in 4M and 7M, but significantly abundant in I_9_-10M (Table 3). One cytoplasmic aconitate hydratase also showed a different pattern, being significantly abundant in I_9_-4 and 10M, but not identified in 7M. Similar results were obtained for two cytochrome c oxidases with one of the proteins showing a major increase in content (13.6-fold-change) and the other 2.6 in I_9_-4M. Both proteins reduced their abundances in I_9_ as the plants aged.

### Proteins Related to Abiotic Stress Response, Photosynthesis, Lignin, and Hormones Biosynthesis

One of the possible consequences of hypoxia is the increased production of reactive oxygen species (ROS) inside the cells and tissues (Bailey-Serres and Voesenek, 2008, 2010; Shingaki-Wells et al., 2014). The ascorbate–glutathione cycle represents an important mechanism for plant cells to avoid the formation of excess ROS. Table 3 shows the identification of five classes of proteins, namely, ascorbate peroxidase, monodehydroascorbate reductase, glutathione reductase, glutathione peroxidase, and glutathione transferase involved in the destruction of ROS. Overall, we observed that the proteins identified showed the highest abundance in I_9_-4M-old-plants. In some cases, such as for glutathione peroxidase and two glutathione transferases, the content of proteins reached 6.7, 8.0, and 20.7-fold-changes. Six proteins identified as glutathione transferases showed a change in their abundance pattern or even a complete absence in I_9_-4M but increasing in the same internode of 7M and 10M-old plants. A peroxidase 2 protein was highly induced (26.3-fold-change) in I_9_-4M and dramatically reduced its abundance as the plant matured to 1.5 in 7M and 0.3 in 10M (Table 3). Another peroxidase 39 precursor was significantly increased by 5.7-fold-change in I_9_-4M and absent in I_9_ of 7M- and 10M-old plants (Table 3).

One important protein involved in O_2_ transport, hemoglobin 2, was highly abundant in I_9_-4M (22.4-fold-change), reducing its content in I_9_ as the internode matured. Two classes of enzymes involved in ROS-scavenging were also found, three isoflavone reductases and two superoxide dismutase highly induced in I_9_-4M (Table 3).

Juvenile internodes are under rapid expansion through cell division and cell expansion, demanding continuous cell wall biosynthesis, remodeling, and lignification. We found three enzymes involved in lignin biosynthesis, namely, cinnamyl ADH, phenylalanine ammonia lyase (PAL), and 4 coumarate-CoA ligase (Table 3). Two proteins of cinnamyl ADH were highly abundant in I_9_-4M, with 3.1 and 13.9-fold-change, whereas a third was only present in 7M, showing a repression in its abundance and was not detected in 4M and 10M. The three PAL proteins identified showed a significant abundance in I_9_-10M and reduction in 7M. One PAL protein showed an increase in abundance (2.2-fold-change) in I_9_-4M. Coumarate-CoA ligase showed an 8.8-fold-change in abundance in I_9_-4M, reduced amount in 7M, and a 2.0-fold-change increase in 10M. Three polyphenol oxidases were also identified, showing a significant increase in content in I_9_-4M-old-plants.

Similar results were observed for proteins forming the light harvesting complexes of PSII in the chloroplasts. Table 3 shows the differences in the abundance of five chlorophyll *a/b* binding proteins, one CP26 and two CP24. With exception of one chlorophyll *a/b* binding protein and one CP24, all proteins were significantly more abundant in I_9_ in the juvenile stages of development (4M and 7M). The variation in fold changes was in the range of 2.6 to 12.8 in 4M, whereas in 7M the values decreased, but were still statistically significant. We also identified two components of the enzyme Rubisco, represented by the small and large subunits. In both cases the proteins were more abundant in I_9_-4M, reducing their content as the plants aged.

The results observed with the identification of proteins related to hormone signaling and biosynthesis also showed a similar pattern as described above, having a higher abundance in I_9_ at the juvenile stages of development (4M). We identified one map kinase involved in the signaling of abscisic acid, two abscisic acid stress-ripening related proteins, and five auxin-induced proteins. In all cases, the proteins showed the higher abundance in I_9_-4M, following a reduction in their content in 7M- and 10M-old plants.

### Metabolites Identified in Developing Culms of 4-Month-Old-Plants

Considering that proteomics revealed a distinct pattern between I_5_ and I_9_ in 4M-old-plants, we decided to investigate whether primary metabolites would have differences in both culms at the same age. In total, 67 metabolites were identified by GC-MS approach (Supplementary Table 7). Most metabolites identified (37%) were classified as “carbohydrates and carbohydrates conjugates,” followed by “amino acids, peptides, and analogs (21%),” and “others” (18%) (Supplementary Figure 3B). To identify the metabolites that most contributed to distinguish between I_5_ and I_9_, we combined VIP scores from PLS-DA (≥1) and *t*-test, FDR adjusted (*p* ≤ 0.05). In total, 27 metabolites were found, with 11 (40%) being differentially abundant (DA) between I_5_ and I_9_, whereas 8 (30%) metabolites were unique for I_5_ or I_9_ (Supplementary Table 8). Organic acids and carbohydrates represented most of the DA or unique metabolites identified, indicating that both classes could be modulated toward culm maturation. Among the organic acids, the intermediates of TCA and related metabolites, cis-aconitate, fumarate, malate and itaconate reduced in I_9_-4M, whereas trans-aconitate was specifically found in I_5_-4M. Ribose-5-phosphate, a key carbohydrate of pentose phosphate pathway, was exclusively found in I_5_-4M.

## Discussion

### Changes in Culm Proteome Reflects the Alterations in the Regulation of Carbon Metabolism During Sugarcane Maturation

Sugarcane has a very efficient C_4_ type of photosynthesis, allowing the synthesis, transport, and accumulation of high concentration of sucrose in the culms. In the same plant, culms have different levels of maturity and sugar content during plant growth. Those located toward the base are in a more advanced stage of maturation compared with those close to the shoot apical meristem. How the proteome in the internodes change during the process of maturation, and how primary metabolism and sucrose accumulation are regulated, is still poorly understood. Label-free proteomics was therefore used to evaluate not only the modifications in protein profile, but also the quantitative changes occurring in the parenchyma cells of internodes. We compared two types of internodes in the same plant, one at an initial stage of development (I_5_) and the other in a more advanced process of maturation (I_9_). The I_9_/I_5_ ratio of each protein identified was used to compare the quantitative changes in proteome of maturing sugarcane culms (I_9_) throughout the plant development in 4M, 7M, and 10M, relative to the juvenile internode (I_5_).

Depending on the growth conditions, the initiation of a new internode in sugarcane occurs, normally, in a period of 5–20 days, and the final elongation occurs between 6 and 8 months (Moore, 1995). The PCA (Figure 1) showed that at 10M the internodes I_9_ and I_5_ are closely related regarding protein profile and abundance. Although the total amount of soluble sugars in I_5_-10M was about half of I_9_ (Table 1), both internodes already reduced the elongation process and were progressing toward complete maturation (Moore, 1995). On the other hand, the main differences between internodes 5 and 9, occurred in the juvenile stages of development, particularly in 4M-old-plants.

The fact that both internodes at 10M had more proteins in common, and the lowest number of unique ones, corroborates the idea that at this stage of development their metabolic status might be similar, being mainly dedicated to sucrose accumulation and maintenance (Watt et al., 2014). The analysis of the distribution of common proteins in both internodes provides further evidence that in the first stages of development (4M), the metabolism is widely different of that at 10M. For instance, in Figure 2 we observed that 85% of the proteins are more abundant in I_9_ whereas only 7% in I_5_ in plants at 4M. These percentages became rather more equilibrated in plants at 7M (32.5 and 34.4%, respectively) and 10M (25.6 and 21.4%, respectively).

The distribution of differentially abundant proteins classified according to their biological process, shown in Figure 3, further demonstrates that the internodes at 10M have the metabolism mainly dedicated to sucrose accumulation, whereas at 4M and 7M the internodes are involved with cell expansion and growth. Such changes in metabolic regulation are also manifested by the differences in classes and number of proteins present, which are widely distinct in both internodes at all plant stages (Figure 3).

### Hypoxic Conditions in Culms at the Early Stages of Development Lead to a Change in Primary Metabolism

Oxygen limitation in plant tissues can occur in several situations such as environmental conditions such as flooding, where the microbiota activity in the rhizosphere is so intensive that the demand for oxygen cannot keep pace with consumption, or in wood-forming tissues of fast-growing trees, such as Eucalyptus (Geigenberger, 2003; Van Dongen et al., 2003; Bailey-Serres and Voesenek, 2008; Budzinski et al., 2016). *In situ* measurements of O_2_ concentration in different plant tissues show that it may be reduced to limiting levels, impairing mitochondrial respiration. For instance, in the stems of *Ricinus communis*, growing under atmospheric conditions, O_2_ concentration varied from 21% (v/v) at the surface of the bark to 7% (v/v) in the vascular tissue, raising the O_2_ concentration to 15% (v/v) in the central parts of the stem (Van Dongen et al., 2003). The bark surrounding sugarcane culms is highly suberized and lignified, and has a high content of wax and lipids, which are impermeable to gas diffusion (Moore and Botha, 2014). The resistance to lateral gas diffusion implies that not only O_2_ is reduced, but also CO_2_ increases, leading to a reduction in mitochondrial respiration (Whittaker and Botha, 1997). Whittaker and Botha (1997) measured the rate of mitochondrial respiration as radiolabeled carbon loss of ^14^CO_2_ upon feeding glucose to internodes I_2_ and I_7_. The authors observed a higher respiration rate in I_2_ compared to I_7_. These results corroborate our proteomic observations as the higher O_2_ availability close to the shoot apical meristem allows I_5_-4M to have a higher respiration rate, compared to I_9_-4M. Four-month-old I_9_ showed significant increases in the abundance of cytoplasmic enzymes involved in sucrose metabolism, glycolysis, ethanolic fermentation, TCA cycle, and hemoglobin 2. The significant increase of hemoglobin 2 in I_9_ is probably a tentative to increase the availability of O_2_ for mitochondrial respiration. Under these conditions, the demand for ATP and NAD^+^ would be provided by the increase in metabolic flux through glycolysis and ethanolic fermentation. The metabolites data showed that TCA intermediates (cis-aconitate and fumarate) and related metabolites (malate and itaconate) were reduced in I_9_-4M, whereas TCA proteins accumulated. This result suggests that TCA metabolites are being rapidly used as substrates by the enzymes to maintain the metabolic flux active under hypoxic conditions. This way, the maturing internodes would be able to rapidly develop and prepare their parenchymatic tissue to accumulate sucrose. Once sucrose reaches its maximum storage capacity in mature culms, the content of enzymes involved in primary metabolism are completely reduced. Figure 4 summarizes all these changes occurring in I_9_-4M, and as the culms matured, sucrose storage increased, starting after 7M and reaching its maximum at 10M.

## Data Availability Statement

The datasets presented in this study can be found in online repositories. The names of the repository/repositories and accession number(s) can be found at: PRIDE repository (https://www.ebi.ac.uk/pride/) under project accession: PXD027410, and the Metabolomics Workbench (https://www.metabolomicsworkbench.org) under project accession PR001185 or directly via Project doi: 10.21228/M8J407.

## Author Contributions

CL designed the study. LB, ML, IB, and TC performed the proteomics works. LF and FM performed the bioinformatics and data bank analysis. All authors wrote the article.

## Funding

This work was financially supported by FAPESP (Ref. Proc. n° 2008/56100-5). Fellowships provided by CAPES to Luis Felipe Boaretto and FAPESP to: IB (Ref. Proc. n° 2012/22227-4) and FM (Ref. Proc. n° 2012/12521-2).

## Conflict of Interest

The authors declare that the research was conducted in the absence of any commercial or financial relationships that could be construed as a potential conflict of interest.

## Publisher's Note

All claims expressed in this article are solely those of the authors and do not necessarily represent those of their affiliated organizations, or those of the publisher, the editors and the reviewers. Any product that may be evaluated in this article, or claim that may be made by its manufacturer, is not guaranteed or endorsed by the publisher.

## Supplementary Material

Supplementary Figure 1 Functional classification of differentially accumulated proteins identified by the PLGS 2.5.1 Software, for (I_5_) and (I_9_), analyzed at 4M **(A)**, 7M **(B)** and after the drought stress at 10M **(C)**, classified according to their differential GoTerm distribution of the Fisher's Exact Test, with FDR < 0.05 and *p* < 0.01 reduced to the most specific term. Percentage of total reference (I_5_–red) and tested (I_9_–blue) protein sequences.

Supplementary Figure 2 Illustration of sugarcane culms (I_5_ and I_9_). Plants average size 110 cm, white bar represents 13.7 cm.

Supplementary Figure 3 **(A)** Partial least square discrimination (PLS-DA) score plot showing clustering of I_5_ and I_9_ samples. R2 = 0.99 and Q2 = 0.86, considering six biological replicates. **(B)** Classification of the 67 metabolites identified was done using HMDB (https://hmdb.ca) and Classfyre (http://classyfire.wishartlab.com).

Supplementary Table 1 Fold changes between I_9_ and I_5_ at (4M) in the abundance of proteins identified by LC-MS^E^. The values of *p* < 0.05 and *p* > 0.95 were considered as statistically significant with FDR ≤ 4% for low or high abundance, respectively, for the I_9_/I_5_ ratio. 4MI5 and 4MI9 represent unique proteins identified for each developmental stage.

Supplementary Table 2 Fold changes between I_9_ and I_5_ at (7M) in the abundance of proteins identified by LC-MS^E^. The values of *p* < 0.05 and *p* > 0.95 were considered as statistically significant with FDR ≤ 4% for low or high abundance, respectively, for the I_9_/I_5_ ratio. 7MI5 and 7MI9 represent unique proteins identified for each developmental stage.

Supplementary Table 3 Fold changes between I_9_ and I_5_ at (10M) in the abundance of proteins identified by LC-MS^E^. The values of *p* < 0.05 and *p* > 0.95 were considered as statistically significant with FDR ≤ 4% for low or high abundance, respectively, for the I_9_/I_5_ ratio. 10MI5 and 10MI9 represent unique proteins identified for each developmental stage.

Supplementary Table 4 Parameters of unique and common proteins with FDR ≤ 4% identified by the PLGS 2.5.1 Software, for (I_5_) and (I_9_), analyzed at 4M.

Supplementary Table 5 Parameters of unique and common proteins with FDR ≤ 4% identified by the PLGS 2.5.1 Software, for (I_5_) and (I_9_), analyzed at 7M.

Supplementary Table 6 Parameters of unique and common proteins with FDR ≤ 4% identified by the PLGS 2.5.1 Software, for (I_5_) and (I_9_), analyzed at 10M.

Supplementary Table 7 All metabolites identified in I_5_ and I_9_ by GC-MS analysis. Metabolite classification was done using HMDB (https://hmdb.ca) and Classfyre (http://classyfire.wishartlab.com) websites.

Supplementary Table 8 Differentially abundant metabolites (VIP ≥ 1.0 and FDR adjusted *p* ≤ 0.05) identified between sugarcane I_5_ and I_9_ culms. Metabolites specifically found in one of the culms (I_5_ or I_9_) are described as Unique.
