# Supplementary material for: Proteomics Reveals an Increase in the Abundance of Glycolytic and Ethanolic Fermentation Enzymes in Developing Sugarcane Culms During Sucrose Accumulation
Source: Front Plant Sci. 2021 Sep 30;12:716964. doi: 10.3389/fpls.2021.716964 (PMC8515036; doi:10.3389/fpls.2021.716964)
Supplement: Supplementary Figure 1 — Functional classification of differentially accumulated proteins identified by the PLGS 2.5.1 Software, for (I5) and (I9), analyzed at 4M (A), 7M (B) and after the drought stress at 10M (C), classified according to their differential GoTerm distribution of the Fisher's Exact Test, with FDR < 0.05 and p < 0.01 reduced to the most specific term. Percentage of total reference (I5–red) and tested (I9–blue) protein sequences. [file Data_Sheet_1.zip › Suppl_Figure_2.PDF]

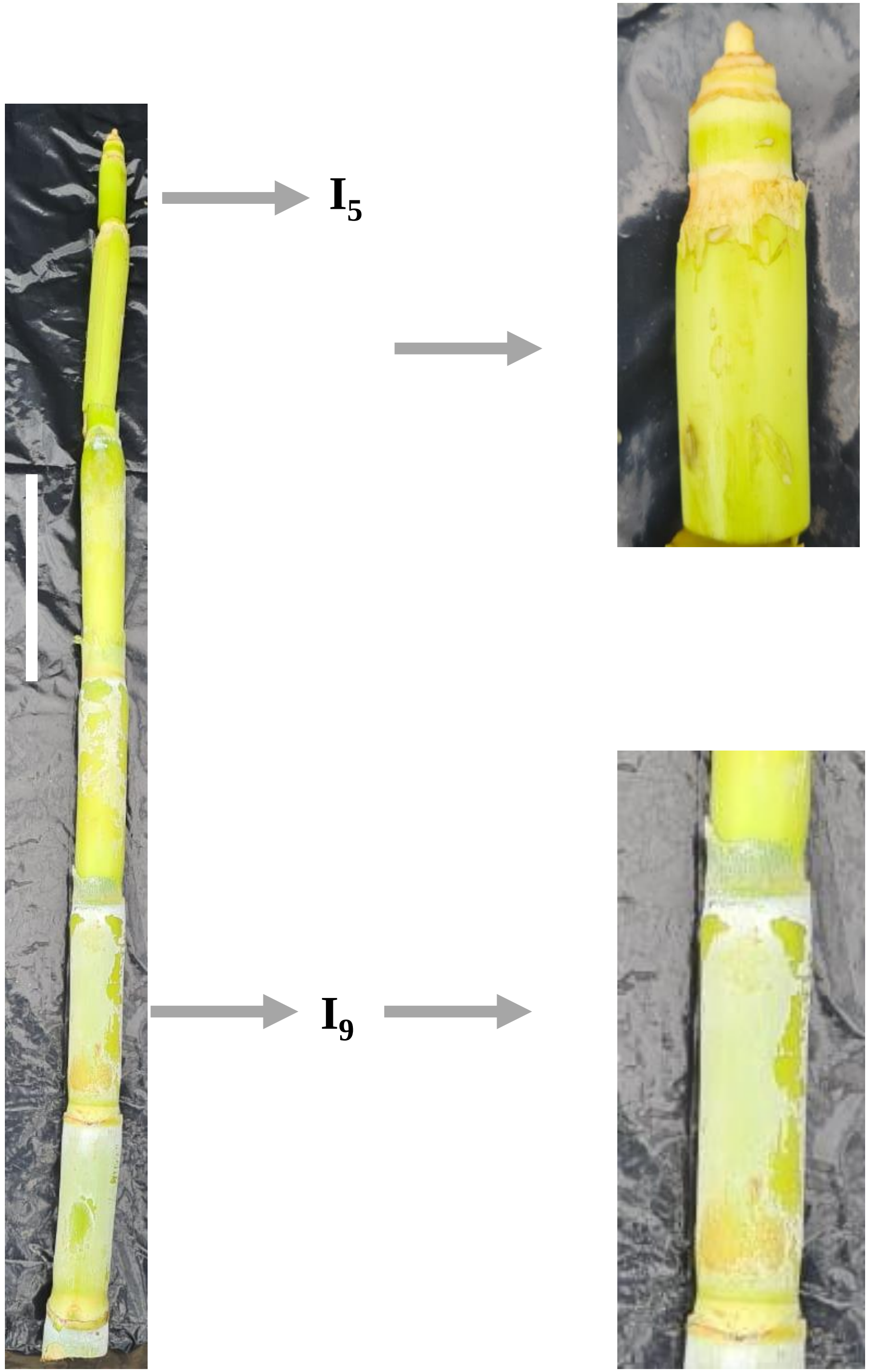

Supplementary Figure S2. Illustration of sugarcane culms (I_5 and I_9). Plants average size was 110 cm, white bar represents 13.7 cm.
